# Molecular characterization and complete genome sequence of avian paramyxovirus type 4 prototype strain duck/Hong Kong/D3/75

**DOI:** 10.1186/1743-422X-5-124

**Published:** 2008-10-20

**Authors:** Baibaswata Nayak, Sachin Kumar, Peter L Collins, Siba K Samal

**Affiliations:** 1 Virginia-Maryland Regional College of Veterinary Medicine, University of Maryland, College Park, Maryland, USA; 2 Laboratory of Infectious Diseases, National Institute of Allergy and Infectious Diseases, Bethesda, USA

## Abstract

**Background:**

Avian paramyxoviruses (APMVs) are frequently isolated from domestic and wild birds throughout the world. All APMVs, except avian metapneumovirus, are classified in the genus *Avulavirus *of the family *Paramyxoviridae*. At present, the APMVs of genus *Avulavirus *are divided into nine serological types (APMV 1–9). Newcastle disease virus represents APMV-1 and is the most characterized among all APMV types. Very little is known about the molecular characteristics and pathogenicity of APMV 2–9.

**Results:**

As a first step towards understanding the molecular genetics and pathogenicity of APMV-4, we have sequenced the complete genome of APMV-4 strain duck/Hong Kong/D3/75 and determined its pathogenicity in embryonated chicken eggs. The genome of APMV-4 is 15,054 nucleotides (nt) in length, which is consistent with the "rule of six". The genome contains six non-overlapping genes in the order 3'-N-P/V-M-F-HN-L-5'. The genes are flanked on either side by highly conserved transcription start and stop signals and have intergenic sequences varying in length from 9 to 42 nt. The genome contains a 55 nt leader region at 3' end. The 5' trailer region is 17 nt, which is the shortest in the family *Paramyxoviridae*. Analysis of mRNAs transcribed from the P gene showed that 35% of the transcripts were edited by insertion of one non-templated G residue at an editing site leading to production of V mRNAs. No message was detected that contained insertion of two non-templated G residues, indicating that the W mRNAs are inefficiently produced in APMV-4 infected cells. The cleavage site of the F protein (DIPQR↓F) does not conform to the preferred cleavage site of the ubiquitous intracellular protease furin. However, exogenous proteases were not required for the growth of APMV-4 in cell culture, indicating that the cleavage does not depend on a furin site.

**Conclusion:**

Phylogenic analysis of the nucleotide sequences of viruses of all five genera of the family *Paramyxoviridae *showed that APMV-4 is more closely related to the APMVs than to other paramyxoviruses, reinforcing the classification of all APMVs in the genus *Avulavirus *of the family *Paramyxoviridae*.

## Background

The family *Paramyxoviridae *contains a large number of viruses of humans and animals [1]. These viruses have been isolated from many species of avian, terrestrial and aquatic animals worldwide. The members of this family includes many human pathogens such as measles (MeV), mumps (MuV) and human respiratory syncytial virus (hRSV) and many important animal pathogens such as Newcastle disease virus (NDV), canine distemper (CDV) and rinderpest (RPV) [2]. Some of the members of the family *Paramyxovirida*e are well characterized, while characteristics for other members of this family remain unknown. Members of this family are enveloped viruses possessing a non-segmented negative-strand genome [1] and are divided into two subfamilies; *Paramyxovirinae *and *Pneumovirinae*. Subfamily *Paramyxovirinae *is divided into five genera: *Rubulavirus *[MuV, human parainfluenza viruses (hPIV) -2 and -4, simian virus type 5 (SV5) and Tioman virus (TiV)], *Respirovirus *[Sendai virus (SeV) and hPIV-1 and -3], *Henipavirus *[Hendra virus (HeV) and Nipah virus (NiV)], *Morbillivirus *[MeV, CDV and RPV], and *Avulavirus *[avian paramyxovirus (APMV) serotypes 1–9]. Subfamily *Pneumovirinae *is divided into two genera: *Pneumovirus *(hRSV and its animal counterparts including bovine respiratory syncytial virus [bRSV]), and *Metapneumovirus *[comprising human metapneumovirus (HMPV) and avian metapneumovirus (AMPV)] [1,3,4].

The genomes of the paramyxoviruses vary in length from 13–19 kb and contain 6–10 genes encoding up to 12 different proteins. Transcription begins at single promoter at the 3' leader end and the genes are copied into individual mRNAs by a start-stop-restart mechanism guided by conserved gene-start and gene-end transcription signals that flank the individual genes [1]. Genome replication involves the synthesis of a complete positive-sense copy of the genome that is called the antigenome and serves as a template for producing progeny genomes. All members of family *Paramyxoviridae *encode a nucleoprotein (N), a phosphoprotein (P), a matrix protein (M), a fusion protein (F), an attachment protein called the hemagglutinin (H) or haemagglutinin-neuraminidase (HN) or glycoprotein (G), and a large polymerase protein (L) [1,2].

All APMVs have been classified into nine different serotypes based on HI test and all NDV strains belong to APMV serotype 1 [5]. Since NDV can cause severe disease in chickens, APMV-1 is the most extensively characterized serotype of the APMVs. Very little is known about the molecular and biological characteristics and pathogenicity of APMV serotypes 2–9. APMV types 2, 3, 6 and 7 have been associated with disease in domestic poultry [6-10]. The APMV-5 (Kunitachi virus) isolated from budgerigar is known to cause disease in wild birds [11]. Other serotypes, including APMV-4, -8, and -9, have been isolated from ducks, waterfowls, and other wild birds with no clinical signs of disease [5,12-15]. The strain duck/Hong Kong/D3/75, isolated from a duck in Hong Kong in 1975, was found to be representative of a distinct serotype of APMVs [16], later designated as APMV serotype 4 on the basis of HI and neuraminidase inhibition (NI) tests [17]. Experimental infection of chickens with APMV-4 and APMV-6 showed mild interstitial pneumonia, catarrhal tracheitis, and BALT or GALT hyperplasia, suggestive of viral disease [18].

An understanding of the molecular and biological characteristics of APMV -2 to-9 is of general interest and is important for developing vaccines and diagnostic tests against these viruses. To date, the complete genome sequence for representatives of APMV-1 [19], APMV-2 [20], APMV-3 [21] and APMV-6 [22] are available. As a first step towards understanding the molecular biology and pathogenicity of APMV-4, we have determined the growth characteristics and complete genome sequence of the APMV-4 prototype strain duck/Hong Kong/D3/75 (GenBank accession no. FJ177514). Previously, sequence was available only for the APMV-4 HN gene (GenBank accession no. D14031). The sequence of the strain duck/Hong Kong/D3/75 was compared with those of other APMV serotypes and other paramyxoviruses in order to determine phylogenetic relationships.

## Methods

### Virus and cells

The APMV-4 prototype strain, duck/Hong Kong/D3/75 was obtained from National Veterinary Services Laboratory, (Ames, IA). The chicken embryo fibroblast (DF-1), Madin-Darby Canine Kidney (MDCK), human epidermoid carcinoma (HEp-2), Baby Hamster Kidney (BHK 21), Bovine Turbinate (BTu), Pig Kidney (PK15), Quail fibrosarcoma (QT35), Rabbit Kidney cells (RK13), African green monkey kidney (Vero), Madin-Darby Bovine Kidney (MDBK), and duck embryo (CCL-141) cell lines were obtained from the American Type Culture Collection (ATCC, Manassas, VA), and turkey embryo fibroblast (TEF) primary cells were made in our laboratory. The DF1 and QT35 cells were grown in Dulbecco's minimum essential medium containing 10% fetal calf serum, while the other cells were grown in Eagle's minimum essential medium containing 10% FCS, at 37°C with 5% CO_2_.

### Virus propagation and requirement of exogenous protease

The APMV-4 prototype strain, duck/Hong Kong/D3/75 was propagated in 9 day-old specific-pathogen-free (SPF) embryonated chicken eggs by inoculation through the allantoic cavity route. The titer of virus was determined by hemagglutination (HA) test using 0.5% chicken RBC at room temperature. Virus propagation was carried out in different cell lines either in the absence or presence of exogenous protease in order to determine a suitable cell line for virus growth. The cell culture medium was supplemented with 5% allantoic fluid, or 1–5 μg/ml of acetyl trypsin/ml (Gibco), or 1–5 μg/ml of α-chymotrypsin/ml (Sigma), each of which provided a source of protease for cleavage of the F protein, if necessary. The cell lines supporting viral growth were observed by corroborating cytopathic effects (CPE) in cells and HA titer in cell culture supernatant both before and after freeze-thawing cycles. The ability of the virus to produce plaques was tested in the different cell lines using 1% methylcellulose with and without exogenous protease in the overlay.

### Isolation of viral RNA and determination of genome sequence

Viral genomic RNA was isolated from purified virus, obtained from infected allantoic fluid by discontinuous sucrose gradient centrifugation, using RNeasy mini kit (Qiagen, USA). The genome sequencing was carried out by using HN gene specific primer designed from the published HN gene sequence (GenBank accession no. D14031). A set of consensus primers were designed for gene start (GS), N and L gene by aligning genomes of published APMV-1, APMV-2, APMV-3 and APMV-6 sequences and working consensus primers were mentioned in Table 1. Reverse transcription of viral genome was carried out by GS consensus primer and primer HN4-1758F (Table 1). A portion of the N gene (256 nt) was amplified by using positive sense GS consensus primer and antisense N consensus primer. The F gene end and HN gene start region was also amplified using primer GS consensus and HN4-81 antisense. The HN gene end and L gene start regions were amplified by sense HN4-1758F primer and L consensus A (L-revA) antisense primer. By comparing these two regions APMV-4 specific gene start and gene end (GE) sequences were identified and APMV-4 specific sense primer APMV-4 GS-F and antisense APMV-4 GE-R primers were designed.

**Table 1:** Primers used in the study

GS consensus (sense)	-5'-GAGCAGTAGGAGCGGAA-3'
AN4-1758F (sense)	-5'-CATTCAAGATAGTGCCATTCCTC-3'
NP consensus (antisense)	-5'-GWWSCYAYWCCCATKGCAWA-3'
APMV4-HN4-81 (antisense)	-5'-ACTTCTGTTGACTTCTCTTGGTA-3'
APMV4 GS-F (sense)	-5'-CTAGGGTGGGGAAGG-3'
APMV4 GE-R (antisense)	-5'-CCGTTTTTAATTAAAAA-3'
APMV4-NP4-2F (sense)	-5'-GAAACTTCCCACACATGTACTCCTATGC-3'
APMV4-P-65R (antisense)	-5'-GAGTCTATGATTGCCGATGATGATTC-3'
APMV4-P-550F (sense)	-5'-AACCGGAAAACATTGAACTGGTGGAGTG-3'
APMV4 HN59-81R (antisense)	-5'-ACTTCTGTTGACTTCTCTTGGTA-3'
NSP1 (antisense)	-5'-ATCAAAAGCACC-3'
NSP2 (antisense)	-5'-AGTAGAAACAAGG-3'
NSP3 (antisense)	-5'-ACAAGGGTGAGG-3'
NSP4 (antisense)	-5'-GTTTTTTCTTCTTAA-3'
NSP5 (antisense)	-5'-ATACGGGTAGAA-3'
L-revA (antisense)	-5'-GGNGCRCACATNSWYTCNCKNAC-3'
L-revB (antisense)	-5'-GGNGCRCACATYTGNSWNCKNAC-3'
L-revC (antisense)	-5'-ACYTCYTTYTCYTTNARNSWRTA-3'
L-revD (antisense)	-5'-GTCATYTTNGCRAADATNCKNCC-3'

For subsequent amplification, reverse transcription was carried out by APMV-4 GS-F primer. The PCR product amplified by APMV-4 GS-F and APMV-4 GE-R primer was cloned in pCR TOPO TA vector (Invitrogen). Sequencing of these clones indicated PCR amplification by single APMV-4 GS-F primer covering the P gene (1616 nt- 2671 nt) and L gene (8204 nt-9394 nt) due to presence of a complimentary sequence in cDNA of APMV-4 at this locations. The sequence of remaining N gene was obtained by amplification and sequencing using sense primer NP4-2F and P gene antisense APMV4-P-65R primer. The sequence of M and F genes was obtained by PCR amplification using APMV-4 specific P gene sense (APMV4-P-550F) and HN gene antisense (APMV4 HN59-81R AS) primer. The complete sequence of the L gene was obtained by primer walking using L gene specific forward primer and nonspecific reverse primers (NSP1-5) used in APMV-6 genome sequencing [22] or L gene specific consensus antisense (L-revA, L-revB, L-revC and L-revD) primers designed by APMVs genome sequence alignment (Table 1). For L gene sequencing, 25 L gene specific sense primers were used along with non specific reverse primers. The sequences of the genome termini were determined by 3' and 5' terminus RACE (rapid amplification of cDNA ends) [20,21,23,24]. For 3' RACE, viral genomic RNA was ligated to adapter1 (5'-GGTTTTGCGGTAAAGGTGGAAGAGAAG-3'), using T4 RNA ligase according to the manufacturer's instructions (Invitrogen). The ligated RNA was purified and reverse-transcribed, using a complimentary adapter 2 (5'-CCAAAACGCCATTTCCACCTTCTCTTC-3') primer. PCR amplification was carried out by sense primer adapter 2 and antisense N gene-specific reverse primer (NP4-192aaR -5'-GGCCTCCCCAGAGC CGTCAATGTTG-3') and (NP4-210aaR – 5'-CCAATTGCAAACTGACGATTAAGC-3'). The 5' RACE was carried out by reverse transcription of genomic RNA using L gene specific sense primer (4-PM14464F- 5'-GCGAACCTGGCAGATACATACAAAC-3'). The tailing of purified cDNAs with dCTP was carried out by using terminal deoxynucleotide tranferase (Invitrogen). The cDNAs were then amplified in separate reactions, using an L gene-specific forward primer (4-PM14574F- 5'-AGTAGTCCCCGCTTTCAAC -3') and an anchored G antisense primer.

Sequencing of cloned DNAs or PCR-amplified products were carried out in 3130xl genetic analyzer by using BigDye terminator v 3.1 matrix standard kit and 3130xl genetic analyzer data collection software v3.0 (Applied Biosystem). The entire genome was sequenced at least three times, and at least once from uncloned PCR product, to ensure a consensus sequence.

### P gene mRNA editing

DF1 cells were infected with APMV-4 virus (AF titer- 2^7 ^HAU) at dilution 10^-3 ^per 25 cm^2 ^flask and cells were harvested at 48 hr post infection for RNA isolation. The mRNAs were isolated using mRNA isolation kit (Invitrogen). The viral mRNAs were reverse transcribed using oligo dT primer. The region flanking the putative P gene RNA editing site was amplified by P gene specific primers (APMV4-P50-74F- 5'-CGGCAATCATAGACTCCATACAGC-3' and APMV4-P536-558R- 5'-CAATGTCTCCG GTTGCTTTGTCG-3'). The PCR products were cloned in pCR TOPO TA vector (Invitrogen). The comparison for the presence of P, V or W mRNAs were carried out by analyzing the sequences from positive clones.

### Sequence and phylogenetic tree analysis

Sequence similarity searches were conducted using the basic length alignment search tool (BLAST) from the National Center for Biotechnology Information (NCBI). DNA pair-wise alignment was done using MagAlign (clustalW) in a Lasergene6 software package. Evolutionary relationships were predicted from the multiple nucleotide sequence alignment of whole genomes of the members of the family *Paramyxoviridae*. The phylogenetic tree was generated by ClustalW program of MegAlign. The amino acid sequence homology and divergence between the genera of the subfamily *Paramyxovirinae *were also obtained by clustalW multiple alignment algorithms.

### Database accession numbers

The complete genome sequence of APMV-4 strain duck/Hong Kong/D3/75 have been submitted in GenBank under accession number FJ177514. For comparative analysis different complete genome sequences of subfamily *Paramyxovirinae *were obtained from GenBank. The databank accession number for these complete genome sequence are as follows: subfamily *Paramyxovirinae*; *Avulavirus*: NC_002617 for APMV-1, EU338414 for APMV-2, EU403085 for APMV-3, NC_003043 for APMV-6; *Rubulavirus*: NC_006430 for SV5, NC_003443 for hPIV2, NC_002200 for MuV, NC_004074 for Tioman virus (TiV); *Respirovirus*: NC_003461 for hPIV1, NC_001552 for Sendai virus (SeV), NC_001796 for hPIV3, NC_002161 for bovine PIV 3 (bPIV3); *Henipavirus*: AY988601 for Nipah virus (NiV), NC_001906 for Hendra virus (HeV); *Morbillivirus*: NC_001498 for Measles virus (MeV), NC_001921 for CDV, NC_006383 for Peste des petits ruminants virus (PPRV), NC_006296 for RPV, NC_005283 for Dolphin morbillivirus (DMV); other paramyxovirus: EF646380 for Atlantic salmon paramyxovirus (ASPV), NC_007803 for Beilong virus (BeV), NC_005084 for Fer de Lance virus (FDLV), NC_007454 for J virus (JV), NC_007620 for Menangle virus (MenV), NC_005339 for Mossman virus (MoV), NC_002199 for Tupaia paramyxovirus (TpV), subfamily *Pneumovirinae*; *Pneumovirus*; NC_001989 for bRSV and NC_001781 for hRSV; *Metapneumovirus*: NC_004148 for HMPV, NC_007652 for AMPV.

## Results

### Growth characteristics of APMV-4

APMV-4 strain duck/Hong Kong/D3/75 produced a titer of 2^7 ^– 2^9 ^HA units in 9 day-old embryonated SPF chicken eggs at 4 days post-inoculation (p.i). The APMV-4 strain duck/Hong Kong/D3/75 was able to grow in the MDBK, BHK21, duck embryo, Vero, DF-1 and QT35 cell lines to a titer of 2^4 ^HA units without the addition of exogenous proteases, which is a requirement for efficient F protein cleavage in many paramyxoviruses. The addition of exogenous protease did not give any difference in titer. The typical cytopathic effect (CPE) observed was rounding and detachment of cells. Multicycle growth curves documented virus release at 20, 28 and 36 hr after infections in MDBK, Vero and DF-1 cells, respectively. The virus reached to a maximum titer in cell culture supernatant at 36 hr p.i in MDBK and Vero cells, while 72 hr p.i in DF-1 cells. The virus did not form any visible plaques in all cell lines tested with or without addition of proteases. Mean death time of the APMV-4 strain duck/Hong Kong/D3/75 in embryonated chicken eggs was zero, indicating its avirulent nature for chickens. Electron microscopy of partially purified virus showed that the virus particles were enveloped, pleomorphic but mostly spherical in shape, with a size ranging from 150–250 nm (data not shown).

### Determination of APMV-4 complete genome sequence

**Figure 1:**
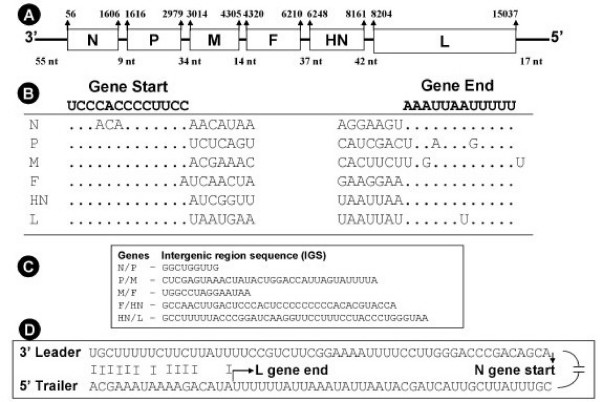
**Schematic diagram of the APMV-4 genome (A) with leader, trailer, gene start, gene end, IGS characteristics. **Alignment of conserved gene start and gene end motifs of the APMV-4 genes (B). Comparison of the variable IGS (C). Complementarities between the 3' leader and 5' trailer regions (D). All sequences are negative sense.

The genome of APMV-4 strain duck/Hong Kong/D3/75 consists of 15,054 nt (GenBank accession no. FJ177514), which follows the "rule of six" common to other members of subfamily *Paramyxovirinae *[25,26]. The genomic organization of APMV-4 is similar to the other members of genus *Avulavirus *in the order of 3'-N-P-M-F-HN-L-5' (Figure 1A). The genes were flanked by leader sequences at the 3' end and trailer sequences at the 5' end. The intergenic sequences (IGS) between genes, which are not copied into mRNAs, varied in length from 9 nt to 42 nt (Table 2). The IGS between N/P, P/M, M/F, F/HN and HN/L are 9, 34, 14, 37 and 42 nt, respectively (Figure 1C). All the genes of APMV-4 were positioned at hexamer phase 2 except F gene that was at hexamer phase 6 (Table 2). The APMV-4 phasing pattern is unique among paramyxoviruses. Analysis of deduced amino acid sequences from open reading frames (ORFs) of all the genes revealed 91.09% coding percentage, which is similar to the coding percentage of other paramyxoviruses [27].

**Table 2:** Genomic features and protein characteristics of APMV-4

Genes	Hexamer phasing at gene start	mRNA characteristics (nt)				Intergenic region (nt)	Deduced protein	
		Length (nt)	5'UTR (nt)	ORF (nt)	3'UTR (nt)		Size (aa)	Mol wt (kDa)

N	2	1551	60	1374	117	9	457	50.03
P/V (P)	2	1364	46	1182	136	34	393	42.02
P/V (V)	2	1365	46	675	644	-	224	23.98
P/V (W)	2	1366	46	414	906	-	137	14.29
M	2	1293	77	1110	106	14	369	41.45
F0	6	1891	74	1701	116	37	566	61.32
F1	-	-	-	-	-	-	446	47.93
F2	-	-	-	-	-	-	120	13.41
HN	2	1914	69	1710	42	42	569	63.08
L	2	6834	95	6636	-	-	2211	249.72

The 3' leader region of APMV-4 is 55 nt, a length that is generally conserved among members of subfamily *Paramyxovirinae *[19]. Comparison of the nucleotide sequence of the APMV-4 leader region with the leader sequences of other paramyxoviruses showed 30–47% percent identity. Surprisingly, the length of the APMV-4 5' trailer region is 17 nt, which is the shortest among the members of the family *Paramyxoviridae *sequenced to date. The sequences of the 3' leader and 5' trailer termini showed a high degree of complimentarity (70.5% complimentarity) for the terminal 17 nt, suggesting the presence of conserved elements in the 3' promoter regions of the genome and antigenome (Figure 1D). The gene-start signal of APMV-4 is 3'-UCCCACCCCUUCC-5' and is exactly conserved among all genes except the N and F genes, in which there are difference of 3 and 1 nt, respectively (Figure 1C). The consensus gene end signal of APMV-4 is 3'-AAAUUAA (U) 5 -5', with difference of 3, 2 and 1 nt in the P, M and L gene, respectively (Figure 1C).

### The Nucleoprotein (N) gene

The APMV-4 N gene is 1551 nt and encodes a N protein of 457 amino acids. It contains a highly conserved sequence motif, 322-FAPGNFPHMYSYAMG-336 (F-X4-Y-X3-α-S-α-AMG, where X is any amino acid and α is any aromatic amino acid), that corresponds to a motif previously identified in the central region of the N protein of other members of *Paramyxovirinae *and which has been implicated in the self-assembly of N with RNA or with other N monomers [1]. Within the conserved motif of APMV-4 N protein, conserved amino acid Y327 is replaced by F327. The N protein of APMV-4 has the highest amino acid identity (51.2%) with that of APMV-3, whereas with other members of *Avulavirus *it shared 34–38% identity. It shared an amino acid identity of 30–35% with *Rubulavirus*, 20–22% with *Respirovirus*, 26–27% with *Henipavirus*, 21–22% with *Morbillivirus*, and 22–25% with unclassified paramyxoviruses (Table 3).

**Table 3:** Percent identity of APMV-4 proteins with the other members of subfamily Paramyxovirinae*.

	**NP**	**P**	**M**	**F**	**HN**	**L**
**Avulavirus**						

APMV-1	34.7	20.2	28.5	32.3	32.9	32.2
APMV-2	38.2	20.2	30.8	33.9	30.6	34.2
APMV-3	51.2	20.5	32.3	33.1	39.2	40.8
APMV-6	38.4	19.8	31.4	32.5	31.6	33.2

**Rubulavirus**						

SV5	30.1	15.2	24.1	27	31.6	31.8
hPIV2	31.4	17.8	23.2	25	30.4	31.7
MuV	32.3	17.3	24.6	27.3	31.6	32.4
TiV	35.8	18.3	21.1	25.6	18.4	31.7

**Respirovirus**						

hPIV1	20.5	6.6	14.6	20.1	22.3	24.1
bPIV3	22.3	7.6	14.6	22	21.2	24.4
hPIV3	22.7	9.1	15.1	22.2	22.3	24.2
SeV	21	5.8	14.6	21.5	22.6	23.4

**Henipavirus**						

HeV	26.6	12.7	15.7	22	15.8	23.9
NiV	27.1	12.2	15.1	21.9	15.3	24.7

**Morbillivirus**						

DV	22.1	11.4	14.6	23.5	13.7	25
MeV	22.3	10.7	16.5	23.1	12.8	24.7
PPRV	22.1	10.2	15.7	21.2	9.5	24.3
RPV	21.4	10.7	15.7	21.9	12.3	25.5
DolMV	22.1	10.7	14.3	23.6	11.2	24.5

**Other paramyxovirus**						

ASPV	22.3	7.1	15.1	24	23.3	23.6
BeV	25.8	11.2	14.3	22.9	23.5	24
FDLV	22.7	8.1	16.2	25	23.5	25.4
JV	24.2	12.4	14.3	24.9	23.2	24.1
MoV	25.5	14.2	16.8	20.8	11.4	24.2
MenV	34.3	17.5	22.2	26.5	18.9	31.7
TpV	24.2	12.4	14.6	21.2	18.2	24.2

* See the Background and Methods for virus name abbreviations.

### The Phosphoprotein (P) gene and P/V/W editing

The P gene is 1364 nt long and encodes a predicted P protein of 393 amino acids. The predicted P protein contains many potential phosphorylation sites with one tyrosine, 11 threonine, and 16 serine residues identified as possible sites using Net Phos 2.0 software. The APMV-4 P protein amino acid identity varies from 19–20% with members of *Avulavirus*, 15–18% with *Rubulavirus*, 5–9% with *Respirovirus*, 12% with *Henipavirus*, 10–11% with *Morbillivirus *and 7–14% with other paramyxoviruses (Table 3).

**Figure 2:**
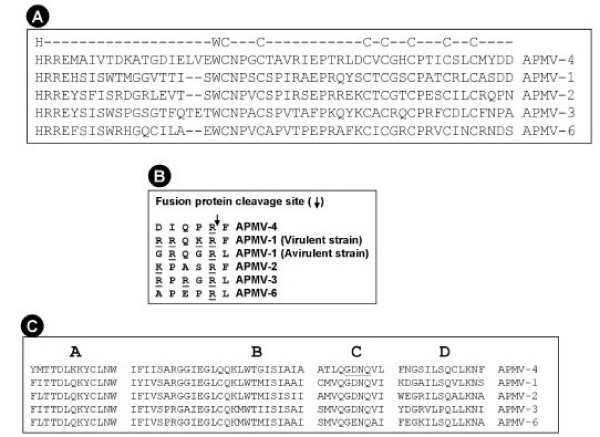
**Amino acid sequence alignment of features of the APMV-4 V, F and L proteins compared with other members of genus *Avulavirus*.** Sequence alignment of V protein C-terminal region (A), F protein cleavage site (B), and conserved domain III of L protein (C).

The P gene contains a putative editing site 5'-AAAGGGGGG-3' (mRNA sense) at positions 442–450 nt of P gene (positions 2057–2065 nt in the complete genome sequence). The insertion of one non-templated G residue would encode a 224 amino acid V protein of molecular weight ~23.98 kDa. This V protein shares its N-terminal 135 amino acids with the P protein. The V-specific C-terminal domain contains seven invariantly spaced cysteine residues and a histidine residue that are highly conserved within paramyxoviruses (Figure 2A). Alternatively, insertion of two non-templated G residues would encode a putative W protein (137 amino acids) comprising the N-terminal 135 amino acids of the P protein and C-terminal two amino acids unique to the W protein.

In order to confirm P gene editing, total mRNA was isolated from APMV-4 infected DF-1 cells. RT- PCR amplification of sequences encompassing the P gene editing site was performed. Sequencing of 28 cDNA clones showed that 18 were unedited, 10 had single G insertion at the editing site, and none had a two G insertion. These results indicated that 35% of the P gene mRNAs from infected cells are edited to encode the V protein and that mRNA encoding the predicted W protein was not detected.

### The Matrix (M) gene

The M gene of APMV-4 is 1293 nt long and encodes a protein of 369 amino acids. The protein is basic in nature with a net charge +12.76 at pH 7.0 and contains 124 hydrophobic amino acid residues. The M protein had an amino acid identity of 28–32% with *Avulavirus*, 23–24% with *Rubula virus*, 20–22% with *Respirovirus*, 15% with *Henipavirus*, 14–16% with *Morbillivirus*, and 14–22% with other paramyxoviruses (Table 3).

### The Fusion (F) gene

The F gene of APMV-4 is 1891 nt in length and encodes a 566 amino acid long F protein. Like other paramyxoviruses, the APMV-4 F protein is a type I integral membrane protein with a C-terminal transmembrane (TM) anchor domain that is predicted to span the host cell membrane. The N-terminus of the F protein contains a signal sequence that mediates translocation of the nascent protein into the lumen of the endoplasmic reticulum and is predicted by the SignalP 3.0 server, to be cleaved between residues 24 and 25 (VHS↓TD). The C terminal TM domain (residues 510–530) anchors the protein in the host cell membrane leaving a short cytoplasmic tail of 32 amino acid residues. In paramyxoviruses, the inactive F protein (F0) becomes biologically active by getting cleaved to F1 and F2 polypeptides by host cell proteases. The F1 and F2 units are joined by disulfide bonding between two cysteines predicted at position C80 of F2 and C346 of F1 unit, according to the cysteine disulfide bonding state and connectivity predictor, DISULFIND [28]. However Iwata et al. [29] provided biochemical data showing that the intra-subunit disulfide bond in Sendai virus was between residue 70, the only residue in the F2 subunit, and 199, the most upstream cysteine in the F1 subunit. The corresponding residues in the F protein of APMV-4 are C80 and C203. The putative F protein cleavage site is DIPQR↓F, corresponding to amino acid positions 116–121 (Figure 2B). Interestingly, the APMV-4 F protein putative cleavage site has a single basic amino acid residue. This resembles the cleavage site of avirulent NDV strains but in contrast to these avirulent strains, APMV-4 was not dependent on exogenous protease for virus growth in cell culture. The N terminus of newly formed F1 subunit of APMV-4 has motif "ALAVAT" (residues 10–15) relative to the F1 N terminus consensus motif "ALGVAT" of most paramyxoviruses. The F protein is a glycoprotein and prediction of potential glycosylation sites by NetNGlyc 1.0 server indicated the presence of one glycosylation site at position 89 in F2 and two at positions 200 and 455 in the F1 protein. In paramyxoviruses, two hydrophobic heptad repeats of the 4-3 pattern are present and are designated HRA and HRB in F1 peptide. Using a prediction server LearnCoil-VMF, similar heptad repeats were detected at position 142–193 as HRA and at position 469–500 as HRB. The deduced amino acid sequence of the APMV-4 F protein has an amino acid identity of 32–33% within *Avulavirus*, 25–27% within *Rubulavirus*, 20–22% with *Respirovirus*, 22% with *Henipavirus*, 21–23% with *Morbillivirus *and 20–25% with other paramyxoviruses (Table 3).

### The Hemagglutinin-Neuraminidase (HN) gene

The HN gene is 1914 nt long and encodes a HN protein of 569 amino acids. Like other paramyxovirus HN proteins, the HN protein of APMV-4 is a type II integral membrane protein that spans the membrane once at its N -terminus and has a predicted hydrophobic signal anchor domain spanning residues 28–46. The HN protein is a glycoprotein and five potential glycosylation sites were predicted at positions 11, 58, 141, 317 and 448 by NetNGlyc 1.0 server. The position 11 is within the predicted signal anchor region and unlikely to be utilized. A putative sialic acid binding motif NRKSCS was found at position 229–234. This protein is acidic in nature with a net charge of -5.696 at pH 7.0. The six conserved neuraminidase active residues were identified at positions 169 (R), 398 (E), 413 (R), 501(R), 529 (Y), 550 (E) along with 11 conserved cysteine residues (167, 181, 193, 233, 246, 339, 460, 466, 470, 534 and 545) corresponding to the globular head of HN protein [30]. Comparison of amino acid homology showed 30–32% identity within *Avulavirus*, 30–31% with *Rubulavirus*, 21–22% with *Respirovirus*, 15% with *Henipavirus*, 9–13% with *Morbillivirus *and 11–22% with other paramyxoviruses.

### The Large polymerase (L) gene

The L gene is 6834 nt long and encodes a L protein of 2211 amino acids. This protein has the highest amino acid identity with APMV-3 (40.8%) as compared to other members of genus *Avulavirus *(32–34%). Deduced amino acid identity varies from 31–32% with *Rubulavirus*, 23–24% with *Respirovirus*, 24% with *Henipavirus*, 24–25% with *Morbillivirus *and 23–31% with other paramyxoviruses (Table 3). The L protein of paramyxoviruses contain six (I-VI) highly conserved linear domains [31], of which subdomain C of domain III is thought to be responsible for transcriptional activity. Domain III is located at positions 644 to 835 and the conserved GDNQ motif of subdomain C is located at position 757–760. The four subdomains (A-D) of domain III are aligned for conserved motifs in Figure 2C. Domain VI contains a highly conserved putative ATP-binding motif K-X18-21-G-X-G-X-G in the subfamily *Paramyxovirinae *[31,32]. A similar conserved motif was found in APMV-4 at position 1767–1793 as motif R-X21-GEGYG with replacement of lysine (K) residue by another basic amino acid arginine (R).

### Phylogenetic analysis

**Figure 3:**
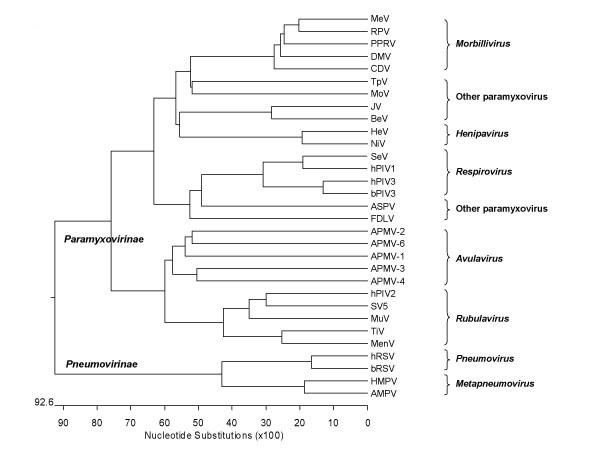
**Phylogenetic tree depicting evolutionary relationship between the members of the family *Paramyxoviridae *based on complete genome nucleotide sequences.** See the Background, Materials and methods for virus name abbreviations.

The phylogenetic relationship of APMV-4 with other members of family *Paramyxoviridae *was obtained by comparing nucleotide sequences of entire genomes. The resulting phylogenetic tree is depicted in Figure 3. The phylogenetic trees were also obtained from percent divergence of deduced amino acid sequences of the N, P, M, F, HN and L proteins (data not shown). In all representative phylogenetic trees, the genera *Rubulavirus, Morbillivirus, Respirovirus, Henipavirus, Avulavirus*, and unassigned paramyxoviruses were separated into distinct clusters. The APMV-4 virus proteins were also clustered within genus *Avulavirus *(data not shown). Within genus *Avulavirus*, APMV-4 showed close evolutionary relationship with APMV-3 and branched together in all the trees drawn from both nucleotide and amino acid distance matrices.

## Discussion

The APMVs are commonly isolated from a wide variety of avian species and are represented by nine serological types. The molecular characterization and pathogenicity of these viruses are mostly unknown except for APMV-1. It is important to determine the molecular characteristics of these viruses. Here, we have characterized APMV-4 strain duck/Hong Kong/D3/75 and determined its complete genome sequence. APMV-4 strains have been isolated from ducks and geese during surveillance studies [17]. We have determined the pathogenicity of APMV-4 strain using MDT test in embryonated chicken eggs. Our result indicated its avirulent nature in chickens. Pathogenicity of APMV-4 in its natural host and in other avian species is yet to be characterized by experimental infections. Experimental infection of chickens by APMV-4 had shown to cause mild interstitial pneumonia and catarrhal tracheitis indicating its disease potential [18]. The growth curve studies in cell culture showed that APMV-4 was able to grow in cells of different species of origin, indicating a broad host range for the virus. The addition of exogenous protease had no effect on the kinetics of virus growth, yield, lack of ability to plaque, or formation of CPE.

The genome of APMV-4 is 15,054 nt long, which is larger than APMV-2 (14,904 nt) [20] and smaller than APMV-3 (16,272 nt) [21]. The typical genome size of members of family *Paramyxoviridae *is approximately 15,500 nt, but can be considerably larger, as exemplified by HeV (18,234 nt) and BeV (19,212 nt) [1]. The length of the APMV-4 genome follows the "rule of six" that is a characteristic of subfamily *Paramyxovirinae *[26] but not of the subfamily *Pneumovirinae *[25]. The leader region of APMV-4 is 55 nt in length, which is exactly conserved in length with most other members of subfamily *Paramyxovirinae *[1]. In most members of family *Paramyxoviridae*, the 5' trailer region is 25–60 nt long [33]. Interestingly, the trailer region of APMV-4 is 17 nt in length, which is the shortest among members of subfamily *Paramyxovirinae *whereas APMV-3 has the longest trailer sequence (707 nt) within the family reported to date [21]. The terminal sequences of the 3' leader and 5' trailer regions showed a high degree of complimentarity (70.5%), suggesting conserved elements in the 3' promoter region of genome and antigenome. Identification of conserve gene start (3'-UCCCACCCCUUCC-5') and gene end (3'-AAAUUAAUUUUU-5') sequences revealed six genes in the order of 3'-N-P/V-M-F-HN-L-5' within the genome with variable IGS. Six genes are also found in all members of the subfamily *Paramyxovirinae *with the exception of presence of the SH gene in APMV-6 of *Avulavirus*, SV5, MuV of *Rubulavirus*, J virus, BeV, and U gene in FDLV [1,34]. The variable IGS of APMV-4 is similar to that of *Rubula, Avula, Morbilli *and *Henipa *viruses, whereas, conserved trinucleotide GUU is typical of *Respirovirus *[33]. The subunit hexamer phasing positions for the start sites of the six genes and the P-gene editing site, shown to be genus-specific within the subfamily *Paramyxovirinae *[32,35]. The hexamer phasing (2, 2, 2, 6, 2, 2) of APMV-4 showed differences with those of the other APMVs [APMV-1(2,4,4,4,3,6), APMV-2 (2,2,2,3,3,3), APMV-3 (2,5,5,2,2,1) and APMV-6 (2,2,2,2,2,4,4)]; suggesting its uniqueness within the members of genus *Avulavirus*.

The N protein core conserved motif of APMV-4 is F-X4-F-X4-SYAMG and a similar motif has also been reported for APMV-2 [20]. This is different from earlier motif F-X4-Y-X4-SYAMG that was reported for other members of *Paramyxovirinae *and is thought to be required for NP-NP interaction. The first amino acid residue F322, needed for correct self-assembly is conserved in APMV-4 as reported for other members of the family [36]. APMV-4 edits the P gene from P to V in a manner similar to that employed by *Avulavirus*, *Respirovirus *and *Morbillivirus*, but differs from that of *Rubulavirus*, which edit their mRNA from V to P [37]. Insertion of one non-templated G residue at the editing site produces the V protein with conserved histidine and seven cysteine residues. The W protein produced by mRNA editing with a double G residue insertion is either absent or rare, as W mRNAs were not detected in cells infected with APMV-4. The M protein is the most abundant structural protein in the virion, and it associates with membranes and with the hydrophobic tails of viral F and HN proteins [1]. The M protein of APMV-4 is rich in hydrophobic amino acids sufficient for hydrophobic interaction but lacks the membrane spanning domain.

The paramyxovirus F protein becomes biologically active when the inactive precursor (F0) is cleaved into disulfide-bonded F1–F2 subunits by host protease [1]. The F protein cleavage site is a well-characterized determinant of NDV pathogenicity in chickens. Virulent NDV strains typically contain a polybasic cleavage site (R-X-K/R-R↓F) that is recognized by furin-like intracellular proteases that are ubiquitous in most cells. This provides for efficient cleavage in a wide range of tissues, making it possible for virulent strains to spread systemically. In contrast, avirulent NDV strains typically have one or a few basic residues relative to the cleavage site and depend on secretory proteases (or, in cell culture, added protease) for cleavage. This limits the replication of avirulent strains to the respiratory and enteric tracts where the secretory proteases are found. However, the cleavage site of wild type SeV F protein (VPQSR↓F) has a monobasic residue (underlined) that limits replication to the respiratory tract, but variants with mutation from serine to proline (PR↓F) showed pan-tropism [38], in addition they do not require exogenous protease for growth of virus. The APMV-4 cleavage site (DIQPR↓F) is similar to efficiently cleaved PR↓F variant of SeV in having a proline immediately upstream of the single arginine residue and also does not require exogenous protease for growth in cell culture. However, the APMV-2 F protein cleavage site (KPASR↓F) contains a SR↓F site similar to that of wild type SeV but does not require exogenous protease for cleavage activation [20]. At the F protein cleavage site, (R↓F) is common in APMV-4, APMV-2 and virulent APMV-1 (RRQKR↓F) irrespective of basic amino acid numbers and does not need exogenous protease for virus growth. In contrast, the F protein cleavage site (R↓L) is common in avirulent APMV-1 (GRQGR↓L), and APMV-3 (RPRGR↓L) irrespective of basic amino acids and require secretory/exogenous proteases for virus growth.

Like other paramyxoviruses, the HN protein of APMV-4 is a type II integral protein with a conserved sialic acid binding motif (NRKSCS) and conserved neuraminidase active site residues and cysteine residues corresponding to the globular head of HN protein [30]. Alignment of the APMV-4 L protein subdomain C of domain III (Figure 2C) with other APMVs showed the conserved catalytic motif GDNQ. Interestingly, in APMV-6, this conserved motif contains a single amino acid difference (GENQ, difference underlined). In the rabies virus L protein, mutation of GDNQ to GENQ abolished polymerase activity in vitro [39]. The APMV-4 L protein had a conserved ATP-binding motif (R-X21-GEGYG) at domain VI, which is similar to that of APMV-3 (R-X21-GEGSG), but for other members of *Paramyxovirinae *this motif is K-X18-21-G-X-G-X-G [31,32].

The phylogenetic analysis of APMV-4 with members of the family *Paramyxoviridae *showed that APMV-4 was more closely related to other APMV types than with other paramyxoviruses, supporting classification of APMVs in the genus *Avulavirus*. APMV-4 showed close evolutionary relationship with APMV-3 both in nucleotide and amino acid analysis. It will be interesting to study further the pathogenicity of APMV-4 in different avian species. The analysis of additional strains of APMV-4 and the development of a reverse genetic system will be helpful to study the molecular biology and pathogenesis of the virus.

## Competing interests

The authors declare that they have no competing interests.

## Authors' contributions

BN carried out the molecular characterization, genome sequencing studies and drafting of the manuscript. SK participated in genome sequencing of the virus. PC participated in design of experiment and manuscript preparation. SKS conceived of the study, and participated in its design and coordination. All authors read and approved the final manuscript.
